# Healthcare Providers' Knowledge of Disordered Sleep, Sleep Assessment Tools, and Nonpharmacological Sleep Interventions for Persons Living with Dementia: A National Survey

**DOI:** 10.1155/2014/286274

**Published:** 2014-03-17

**Authors:** Cary A. Brown, Patricia Wielandt, Donna Wilson, Allyson Jones, Katelyn Crick

**Affiliations:** ^1^Department of Occupational Therapy, University of Alberta, 2-64 Corbett Hall, Edmonton, AB, Canada T6G 2G4; ^2^Occupational Therapy, Central Queensland University, Building 6, Bruce Highway, Rockhampton, QLD 4702, Australia; ^3^Faculty of Nursing, Edmonton Clinic Health Academy, University of Alberta, 87 Avenue, Edmonton, AB, Canada T6G 1C9; ^4^Department of Physical Therapy, University of Alberta, 3-44C Corbett Hall, Edmonton, AB, Canada T6G 2G4

## Abstract

A large proportion of persons with dementia will also experience disordered sleep. Disordered sleep in dementia is a common reason for institutionalization and affects cognition, fall risk, agitation, self-care ability, and overall health and quality of life. This report presents findings of a survey of healthcare providers' awareness of sleep issues, assessment practices, and nonpharmacological sleep interventions for persons with dementia. There were 1846 participants, with the majority being from nursing and rehabilitation. One-third worked in long-term care settings and one-third in acute care. Few reported working in the community. Findings revealed that participants understated the incidence of sleep deficiencies in persons with dementia and generally lacked awareness of the relationship between disordered sleep and dementia. Their knowledge of sleep assessment tools was limited to caregiver reports, self-reports, and sleep diaries, with few using standardized tools or other assessment methods. The relationship between disordered sleep and comorbid conditions was not well understood. The three most common nonpharmacological sleep interventions participants identified using were a regular bedtime routine, increased daytime activity, and restricted caffeine. Awareness of other evidence-based interventions was low. These findings will guide evidence-informed research to develop and test more targeted and contextualized sleep and dementia knowledge translation strategies.

## 1. Introduction

Disordered sleep (DS) is defined as a range of sleep problems including hypersomnia conditions (such as sleep apnea and narcolepsy), parasomnia conditions (such as confusional arousal, restless leg syndrome, and sleep walking), insomnia, and sleep-wake cycle disturbances [1]. All of these DS conditions share the outcome of nonrestorative sleep. Disordered sleep in older persons is a largely overlooked contributing factor for psychosocial dysfunction, decreased cognitive function, depression, agitation, numerous health problems [2], and a major reason for institutionalization in later life. The relationships between DS and decreased cognitive, emotional, and physical functioning, substance misuse, and various mental health problems are well documented [3]. Research suggests that this is a bidirectional relationship such that poor sleep influences health and dementia, and vice versa. This presents an exciting proposition because interventions for DS may reduce the risk for, or lessen the severity of, mental and physical health problems. Promoting better sleep can therefore contribute to health, wellbeing, and continued independent community living for persons with dementia (PWD).

Older adults, and particularly PWD, are at significant risk for altered sleep patterns and DS [4]. Disordered sleep is both a consequence of poor health and a risk factor for the onset of various mental and physical health conditions. For example, animal studies have demonstrated a relationship between sleep deprivation of only three weeks and accelerated development of the amyloid plaques associated with Alzheimer's disease [5]. Other researchers have revealed clear links between insomnia and cognitive tasks such as vigilance [6], concentration, memory, and executive function [7, 8].

The relationship between DS and a range of health conditions common in older persons has been demonstrated. Traumatic brain injury [9], Parkinson's disease [10], and stroke/CVA [11], for example, carry an increased risk of reduced or altered cognitive functioning. Adding DS contributes an additional risk to cognitive decline and the increased likelihood of reduced independence, deteriorated quality of life, and increased caregiver/family burden. DS is a significant predictor of depression among well community-dwelling older adults [12] and, cyclically, depression is a risk factor for dementia [12]. Disordered sleep is also an issue for family caregivers. Providing home-based care to PWD can significantly affect the sleep of family members, and therefore their health and ability to cope with the emotional and physical demands of caregiving [4], all to the detriment of their continued ability to maintain the PWD at home. Support for caregivers is now understood as key for preventing institutionalization [13].

Healthcare providers, service organizations, and care providers lack awareness regarding DS and sleep interventions for both PWD and for their sleep-deprived caregivers [14]. Although nonpharmacological sleep interventions (NPSIs) are effective for improving restorative sleep among older persons [15, 16], the inaccurate belief that reduced hours of sleep and decreased ability to sleep well in old age are “normal” aspects of aging is pervasive [17]. This mistaken belief on the part of both healthcare providers and the general public, coupled with both PWD and their family members reluctance to seek help for sleep issues, contributes to the underdiagnosis and undertreatment of DS in this high need and growing population.

The survey goal was to identify HCP's sleep and dementia evidence-to-practice gaps so as to guide development of knowledge translation (KT) strategies and, ultimately, facilitate better management of DS in PWD. Specifically we surveyed healthcare providers across Canada working in the area of older adult care to identify (1) their level of knowledge related to risk factors for DS in PWD, (2) screening practices and knowledge of sleep assessment tools relevant to DS in PWD, and (3) participants' use of, and perceptions about, the practicality of NPSI for PWD.

## 2. Materials and Methods

### 2.1. Sample

An Internet search, followed by a snowball technique, identified 318 national and provincial Canadian healthcare provider professional organizations and associations. Each was contacted, requesting them to disseminate an online survey to their members. A small number did not respond to repeated emails and presumably did not forward the invitation to their members. Some allowed us access to membership email lists, others disseminated the survey invitation through their internal communication networks, and others forwarded the request to different groups we were unaware of at that time. We estimate that 65–80 organizations participated in distributing the survey invitation. This range of strategies was highly effective for achieving a large and diverse participant group across Canada, but this meant ultimately that we were unable to tally exactly how many healthcare providers were invited to participate. Although we originally intended to have the survey in both the English and French languages, circumstances precluded having a French language survey tool and this report deals only with the English language responses.

### 2.2. Survey

The extant evidence for NPSIs for PWD [1] formed the evidence base for the survey questions. We asked what healthcare providers knew about risk factors for DS in PWD, the consequences of DS, sleep assessment methods, and NPSIs for PWD. We also gathered background demographics. The draft survey was piloted and revised before its distribution. The online survey, approved by the Health Research Ethics Board of the University of Alberta, Edmonton, Canada, and built on the open access software platform “FluidSurvey,” ran between October 2011 and March 2012. The Northwest Territories was not included as regulatory issues precluded surveying in this jurisdiction.

### 2.3. Analysis

The quantitative data received had descriptive statistical analyses done using SPSS (version 19.0). We performed 2-tailed statistical testing, with Bonferroni correction, at the .01 level of significance.

## 3. Results

### 3.1. Demographics and Practice Profile

There were 1,846 survey participants who completed the survey in whole or in part. Participants were not required to answer all of the questions and could make more than one selection for many of the questions. Consequently the total number of participants and responses for each question varied. Participants were able to select multiple practice settings so as to best reflect their actual working patterns. For example, a number of participants indicated they worked both in long-term care (LTC) and in acute care settings. Long-term care (LTC) (33.6%, *n* = 680), followed by acute care (32.2%, *n* = 652), accounted for the majority of work settings. Healthcare providers working in the community appeared to be underrepresented (17.4%, *n* = 351) (Table 1 provides the full breakdown by work setting).

Postal codes were provided by 1052 (57.0%) participants and revealed a cross-Canada distribution with the exception of the Northwest Territories. Alberta (38.7%), Ontario (18.9%), and New Brunswick (14.6%) had the highest rates of participation. Respondents worked primarily in nursing (61.1%), physiotherapy (PT) (10.8%), or occupational therapy (OT) (6.2%). Thirty-nine percent (38.8%) of the participants reported that “50% or greater of their patients with dementia resided in the community.” Similarly, 37.9% reported that “50% or greater of their patients with dementia resided in institutional settings.”

### 3.2. Awareness of Sleep Issues for Persons with Dementia

Of 1526 participants responding to the question “*what percentage of patients with dementia are likely to have sleep problems?,*” 10.7% (*n* = 164) indicated they believe that this was the case for “less than 25%” of their patients, and 42.9% (*n* = 655) indicated “25–50%.” Of the other participants, 30.5% (*n* = 534) indicated they believe “51–75%” of their patients with dementia have sleep problems and 11.3% (*n* = 173) indicated “76–100%”.

Participants reported they most often became aware of sleep problems during their usual assessment practices (32.3%, *n* = 837) or from another member of the team (26.9%, *n* = 699). Few reported they became aware through family or patient reports (18.6% and 15.8%%, resp.). Many selected more than one option for this question indicating that sources of information may be quite individual depending on the patient.

### 3.3. Awareness of Sociocultural and Behavioral Factors Associated with Sleep Problems in Persons with Dementia

Participants were asked to select factors from a total of 16 behavioral and sociocultural factors they believe to be associated with the likelihood of sleep problems in PWD (Table 2). All 16 factors were evidence based with no distractors. The most frequently selected factor was “daytime sleepiness” (88.9%) and least selected factor was “smoking” (1.5%). Awareness of sleep-related factors varied across professional groups but participant knowledge was generally low. Table 2 illustrates for each of the 16 factors which profession had the highest and lowest selection rate, and the percentage of the participants within these two groups making the selection. For example, physicians had the highest selection of the factor “alcohol use” (38.3%), while the lowest selection of this factor was by recreation therapists (5.1%). Table 2 also displays where statistically significant differences were found between the two professions with the highest and the lowest selection rate for each factor. For example, the difference between the percentage of physicians who identified “falls” as related to DS in PWD compared to pharmacists (51.2% and 26.9%, resp.) was statistically significant (*P* = .006).

### 3.4. Awareness of Comorbidities Associated with Sleep Problems in Persons with Dementia

Participants selected from 15 physical comorbidities and one category labeled “mental health” those comorbidities they believed were associated with the likelihood of sleep problems in PWD (Table 3). There were no distractors. Table 3 details which profession had the highest or the lowest selection rate, and what percentage of the participants within these two professional groups made the selection. The most frequently selected comorbid condition was “mental health” identified by 92.9% of psychiatrists and the least frequently selected “allergies” (1.7%) made by recreation therapists. There was high between-group variation in selection. For example, 32.1% of nursing participants selected “gastrointestinal (GI) disorders” as related to poor sleep but only 19.0% of psychiatrists selected this factor. Overall, psychiatrists most often had the highest selection rate for the sixteen factors. Only two of the 16 factors, “mental health” and “pain” (selected by 92.9% and 85.7%, resp., of psychiatrists), were also selected frequently by other participants. However, aside from “urological conditions” (selected by 74.1% of physicians), all other physical comorbidities were selected by less than 65% of any of the members of each profession. Table 3 also displays any statistically significant differences between the two professions with the highest and the lowest selection rate for each factor. The difference between the highest and lowest selection rate was statistically significant for all comorbidities except “allergies,” “gastrointestinal disorders,” and “rheumatic disease.” These three were infrequently selected by all participants.

### 3.5. Experience and Awareness of Standardized Sleep Assessment Tools

Participants were asked to identify sleep assessment tools they had used, ones they were aware of but had not used, and ones they felt were not practical in their practice setting. All of the assessment tools were derived from the evidence base. Three nonstandardized tools, “caregiver report” (selected by 97.2% of social workers), “patient self-report” (92.5% of physicians), and “sleep diaries” (78.9% of psychiatrists), were found as having been used by higher numbers of the participants in each professional group. Of the standardized tools, “polysomnography assessment in a sleep lab” had been most often used, but by only 31.2% of psychiatrists and 24.7% of physicians. Of the other standardized tools, less than 30% of any of respondents in any of the professional groups had used the “Epworth” (28.6% of physicians), the “SDI (Sleep Disturbances Index)” (14.3% of social workers), the “SSS (Stanford Sleepiness Scale)” (6.6% of physicians), the “PSQI (Pittsburg Sleep Quality Index)” (6.2% of psychiatrists), the “MOS-SS (Medical Outcomes of Sleep Study Scale)” (3.7% of recreation therapists), and the “Verran Snyder-Halpern Sleep scale” (2.6% of physicians).

We found minimal experience with sleep scales designed specifically for populations where high rates of dementia would be expected; the “PDSS (Parkinson's Disease Sleep Scale)” had been used by 3.9% of physicians, followed by the “SCOPA (Scales for Outcomes in Parkinson's Disease-Sleep Scale)” which had been used by 1.9% of recreation therapists. “Actigraphy,” although considered in the literature as a reasonably affordable objective measure of a number of sleep parameters [18], was very seldom used (5.0% of the physicians). Full details by profession are available from the author on request.

### 3.6. Use and Perceived Practicality of Nonpharmacological Sleep Interventions

The respondents were also presented with a list of NPSIs derived from the evidence base specific to persons with dementia. Participants were asked to select responses for each in four categories so as to capture the breadth of experience with, and perceptions about, each NPSI. The categories were (1) “*recommended in the past and practical for patients to use*,” (2) “*recommended in the past BUT not practical for patients to use*,” (3) “*not previously recommended but may be practical for patients*,” and (4) “*not recommended and not practical*.” Overall, participants indicated they had minimal experience with most NPSIs listed, with the exception of “increased daytime activity” and “decreased daytime napping.” They were positively disposed to many of the interventions as indicated by participants' rating a number of NPSIs as potentially useful despite having no experience with them. There were four NIPSs that participants selected as having previously used but now believed to be impractical: “increased time outside” (24.0%), “warm bath before bed” (19.6%), “sleep restriction regime” (16.2%), “PWD sets own bedtime” (16.8%), and “adjust caregiver bedtime to that of PWD” (15.2%). Full details by profession are available from the authors on request.

## 4. Discussion

The representativeness of the participants, the implications of findings, and emergent points for action are discussed below.

### 4.1. Representativeness of Survey Participants

Nurses comprised 61.1% of the participants, followed by PT (10.8%) and OT (6.2%). This is considered a fairly representative healthcare professional sample, and particularly for PT and OT, because only 11.7% (686/5841) of OT members of the Canadian Association of Occupational Therapists (CAOT) report working in the area of older adult rehabilitation (CAOT, 2012). The 113 OTs participants in this survey potentially represent 1 in 6 of these therapists. For PT, their national organization reports that 5.6% (574/10,253) of their members are identified as working in the area of older adult rehabilitation. The 199 PTs participants in this survey potentially represent 35%, or 1 in 3, of these therapists (personal correspondence 25/10/2012).

This representation from nurses, PT, and OT is a research strength because these healthcare providers typically have frequent, in some cases weekly or even daily, contact with PWD and their families. As such, they may be best suited to assume a triage and advocacy role to alert other team members to possible DS. The low proportion of participants who were physicians (4.0%), psychiatrists (4.2%), or psychologists (<1%) is unsurprisingly given the scarcity of geriatricians and psychogeriatricians in Canada. The Canadian Academy of Geriatric Psychiatry reported in 2012 that only 217 psychiatrists worked predominantly in geriatrics (personal correspondence 29/10/2012). While membership in this national organization is voluntary, it is presumably indicative of the low number of psychiatrists in Canada who work with persons who have dementia. However, a total of 86 psychiatrists completed a survey, and we cautiously propose this is a sufficiently representative sample from which we can draw some conclusions.

### 4.2. Knowledge of the Prevalence of DS and Risk Factors for DS in PWD

The prevalence of sleep problems in community-dwelling persons with dementia is conservatively estimated at 40% [3, 19]. The prevalence in LTC settings, because of the residents' poorer health, decreased activity levels, noise, light, and temperature that is nonsleep conducive at night, and staffing practices and routines, requiring activity at night, would be higher. It has been proposed that some LTC residents do not get a full hour of sleep in any 24-hour period [20]. Close to one-half of the survey participants estimated that 50% or more of their patients had sleep problems. Given that over 60% of our participants are identified as working in long-term care or other institutional care settings, it appears that they underestimate the prevalence of the problem as presented in the literature.

Knowledge about indicators of risk related to DS, aside from “daytime sleepiness,” was generally low. None of the risk factors were identified by 85% or greater of the respondents within any professional group. Notable deficits in awareness were evident about the relationship between sleep and “appetite,” “falls,” “problem solving,” “alcohol,” “smoking,” and “caregiver beliefs.” In these categories, none of the professional groups had greater than 55% awareness of the relationship between the factor and DS (Table 2) and in some awareness fell to less than 5%. “Smoking” and “caregiver beliefs” were the least frequently selected although the evidence is clear that smoking, which contributes to apnea, is related to poor sleep [21]. Similarly, the influence of caregiver beliefs as influencing DS was underrecognized [22]. It is possible that participants, over half who are working in institutional settings, did not select “smoking” because it is largely controlled by staff in LTC settings. However, previous smoking habits can influence respiratory function and apnea. Caregiver beliefs are very important as they assume increasing responsibility and decision making for their family member with dementia. Healthcare providers who understand caregiver beliefs about sleep can address misunderstandings or values that pose challenges to effective sleep management. It is also notable that awareness of different factors varied across professions such that not one profession appeared to be consistently most aware of the evidence-based link between specific risk factors and DS. This is important because it reinforces current guidelines emerging from knowledge translation (KT) research recommending that targeted educational and KT strategies, building on existing awareness and addressing specific knowledge gaps within individual groups, are likely to be more effective compared to “one-size-fits-all” generic education strategies [23].

### 4.3. Knowledge of the Relationship between Sleep and Other Comorbidities

Overall knowledge about relationships between sleep and evidence-based comorbid health conditions was low. Of particular concern were the low (less than 25% in any professional group) endorsement rates for a relationship between DS in PWD and the comorbid conditions of “allergies,” “endocrine disorders,” “renal disorders,” “rheumatic diseases,” and “sensory deficits.” This lack of knowledge matters because of the growing evidence base supporting a bidirectional, reciprocal relationship between these health conditions and DS in PWD [20, 24]. Sleep problems tend to be underrecognized in PWD as the complexity of comorbidities increases and managing the other aspects of dementia becomes an increasing challenge [4]. It is possible that among the survey respondents comorbid conditions, such as these, which routinely alert HCPs to the possibility of DS in noncognitively impaired populations, tend to be overlooked in PWD.

### 4.4. Awareness of Sleep Problems

Participants became aware of sleep problems either through their usual assessment practices (32.3%) or when reported by another team member (26.9%). Family member reporting was identified less than 20% of the time, so it appears that responsibility for recognizing a potential sleep problem rests with the HCP directly or through a referral from another member of the formal healthcare team. However, it is possible that families do not routinely bring concerns about DS to the attention of any HCP, who then must rely more on their own practice and colleagues. Given these findings and the complexities of detecting DS in PWD, it would be of benefit to identify opportunities to include basic sleep-related screening questions in existing, routinely administered, geriatric assessments. For example, DS is a risk factor for falls [25], and significant resources have been dedicated to falls reduction and prevention programs in many countries. However, the Morse Falls Scale (available at http://www.patientsafety.gov/SafetyTopics/fallstoolkit/media/morse_falls_pocket_card.pdf) and the St. Thomas's Risk Assessment Tool in Falling Elderly Inpatients—STRATIFY [26], two of the most widely used, psychometrically sound falls assessment tools, do not contain any sleep-related questions. A literature search for falls risk assessment tools revealed one in-house tool, the FARAM, developed by Bayside Health in Victoria, Australia (available at http://www.health.vic.gov.au/qualitycouncil/downloads/falls/tools.pdf) that incorporated sleep-related questions. Although not yet validated, the sleep-related questions in the FARAM tool could serve as model. Given the clear evidence-based relationship between falls and sleep disorders [25] embedding basic sleep screening in falls assessment tools seems to be a practical step forward to facilitate busy HCPs' routine sleep screening.

### 4.5. Knowledge and Use of Sleep Assessment Tools

Assessment tool knowledge was limited for any assessments beyond nonstandardized self-report measures such as sleep diaries and caregiver reports. These types of self-report tools, while important, are often insufficient to capture a full picture of the extent and characteristics of DS. Current guidelines recommend a combination of self-report, observational, and standardized tools so as to best understand the complexity of DS in PWD [2]. Encouragingly, none of the tools were perceived as highly impractical by the respondents, with the exception of polysomnography. This is not surprising, given the challenges to ensure a sufficient degree of reliability when using polysomnography with PWD. These findings may indicate that, although awareness and experience with standardized tools were low, HCP saw the relevance of sleep assessment and did not regard these tools as impractical. This is a promising finding and interdisciplinary strategies to deliver education about available sleep assessment are indicated. Sleep assessment tools of particular relevance to PWD include actigraphy [18], the Sleep Disturbance Index (SDI) [4], the Pittsburg Sleep Quality Index (PSQI), and the Epworth Sleepiness Scale (ESS) [27]. There is a scarcity of assessment and screening tools specific to SD in PWD, with scope then for further research and development.

### 4.6. Awareness of, and Experience with, Nonpharmacological Sleep Interventions

Only three of the 22 evidence-based nonpharmacological sleep intervention strategies listed in the survey (i.e., “regular bedtime routine,” “increased daytime activity,” and “restricted caffeine”) were reported as having been recommended and perceived as practical by greater than 80% of the entire sample (86.2, 84.2, and 83.2%, resp.). Four of the remaining interventions were endorsed as practical by between 70 and 79% of participants: “regular exercise routine” (76.0%), “restricted daytime naps” (75.7%), “decreased evening noise levels” (73.9%), and “restricted evening fluids” (70.4%). As such, there was reasonable knowledge of these interventions.

The remaining 15 interventions were selected with much less frequency and with greater between-group variation. “Education about sleep surfaces” was more likely to be recommended by OT and PT than other participants, while “evening warm bath” was endorsed most often by psychiatrists and social workers. “Use of a white noise machine” was most likely to be endorsed by social workers and “reduced ambient lighting at night” was more likely to be endorsed by psychiatrists and physicians. “Sleep restriction regime” was most likely to be endorsed by psychiatrists and physicians were more likely to endorse “increased daytime ambient lighting.” Physiotherapists were less likely to endorse “adjustment of caregiver sleep schedule” and “allowing PWD to self-determine sleep schedule.” While social workers were most likely to endorse “caregiver respite care,” physicians and psychiatrists were more likely to endorse “respite care for PWD.” While we can only speculate as to the reason for these professional-specific differences, it may reflect professional training and differing priorities for healthcare or rehabilitation that would promote experience with, and a preference for, certain interventions over others.

Interestingly, interventions targeting modification to the nighttime sleep environment showed weak endorsement overall. It may be that the significant role that the environment plays in sleep is not well understood. While reduced nighttime light may not have been strongly endorsed by HCP working in LTC settings because of potential safety concerns, white noise machines and modification of sleep surfaces/bedding are relatively pragmatic considerations with no evident safety barriers that would preclude selection of these interventions by LTC workers.

It is noteworthy that only 44.6% of participants overall had knowledge of and endorsed “increased time outdoor” while other interventions such as “increased exercise” (76.0%) and “relaxation techniques” (67.5%) were identified much more frequently as having been tried and as also being practical. This apparent preference for active interventions may reflect the current social trend to view exercise as a form of medication for many conditions (e.g., http://exerciseismedicine.org/). Additionally, a lack of awareness among the respondents overall was noted about the significant role exposure to daylight plays in sleep regulation. It is possible that the high number of participants who report working in LTC and other institutional settings may have skewed the findings since daylight exposure is a much less realistic option in these settings compared to PWD living with family caregivers in the community.

### 4.7. Emerging Needs and Recommendations

Box 1 outlines ten recommendations to increase healthcare providers' awareness of sleep and dementia that emerge from our discussion of the survey findings. Recommendations include the need for capacity building across HCP groups to help them better understand the factors that influence sleep, the range of evidence-based risk factors, and the relationship between insufficient sleep and comorbidities. Additional recommendations address HCPs' need for more information about assessment tools and NPSI strategies—particularly pragmatic, evidence-based, environmental modifications. Because homecare nurses, OTs, and PTs have the most extended and frequent contact with PWD and their families, they may be best suited to a sleep triage role. We recommend that these HCPs should be a priority for more in-depth education. Other recommendations, informed by KT best-practice guidelines, highlight that KT strategies should be strength based, target clear knowledge gaps, include family members and other stakeholders when possible, and address the importance of family caregiver beliefs and values about sleep. Final recommendations focus on the cross-disciplinary need for understanding the foundational physiological role that light exerts on sleep, the need to educate both individual HCPs and organizational decision-makers so that sleep-friendly sustainable environments and routines are created, and the importance of contextualized information delivered with attention to the audiences' preferred learning styles.

An evidence-informed approach to HCPs' unmet need for sleep and dementia knowledge is important. Lessons from the examples of KT initiatives in diabetes, cardiovascular care, medication adherence, and guideline implication [23, 28, 29] will help us move forward more effectively in addressing this challenge. Knowledge translation evidence, applied to the survey findings, reinforces the need to plan dissemination strategies addressing demonstrated knowledge gaps and building on existing awareness within individual professional groups. This KT approach, as opposed to delivering less targeted, generic sleep education, can help address HCPs' need for learning that accommodates time constraints. Targeting identified knowledge gaps increases the relevance of information to the recipient. Building on existing knowledge that is already familiar and comfortable for the learner helps achieve knowledge user engagement and perceived self-efficacy with new information [28]. Elwyn et al. [28] propose that KT research neglects the important elements of communication theory and the evidence generated in the field of business and marketing. Drawing from these fields they developed the “sticky knowledge” conceptual model which suggests that the existence of ambiguity and uncertainty, the degree of credibility of both the source of information and the information itself, the learners' absorptive and retentive capacity for new information, the contextual elements influencing the organization's receptivity, and the existence of challenging arduous relationships will all influence the flow of information between sender and receiver. Importantly, these factors also influence how well the information “sticks” to the receiver and then, in turn, flows to the next recipient. As in other KT models, the importance of contextualized information so that the relevance is clearly evident to the recipient is highlighted. These concepts can be applied to help refine KT strategies addressing HCPs' gaps in sleep and dementia knowledge and practice.

*Summary.* These findings indicate that awareness is generally low and knowledge varies between professional groups. This again highlights that, consistent with examples of KT in other areas with evidence-to-practice [23], sleep and dementia awareness and intervention strategies may benefit from targeting specific professions and their identified knowledge gaps instead of focusing efforts into more generic sleep educational approaches intended for all service providers.

*Limitations.* The survey had several limitations. The sample was one of convenience and we have no certainty that it was representative. Additionally, because participants were able to select more than one response for some questions, we cannot conclude that responses were rank ordered. Losing our ability to complete all aspects of the survey in French as well as English language leaves Francophone HCPs working with PWD underrepresented. Finally, and most importantly, healthcare providers working with PWD living in the community were also underrepresented. This is a critical setting for attention as effective and early sleep intervention in the community can act as a preventative measure to support caregivers' wellbeing, optimize PWD's functional capacity, and possibly reduce the risk of institutionalization.

## 5. Conclusions

At the start of the study we knew from clinical experience and from the research literature that sleep problems for PWD were largely unrecognized and often undertreated. As a consequence of this survey, we now know that HCP groups have different knowledge strengths and knowledge gaps. These findings will guide future, evidence-informed, research to develop and test the outcomes of more targeted and contextualized sleep and dementia KT strategies.

## Figures and Tables

**Box 1:**
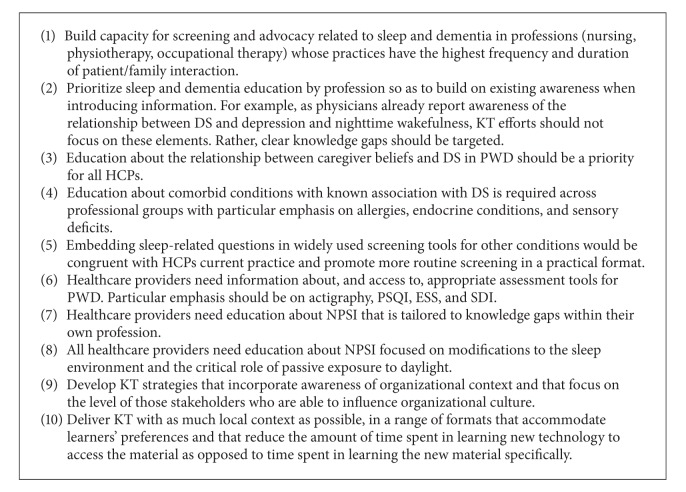
Action points for NPSI KT strategies.

**Table 1:** Practice setting.

Long-term care facility	33.9% (616)
Acute care facility	33.1% (602)
Community/homecare service	16.3% (295)
Family practice/primary care	8.8% (159)
Rehabilitation service	8.5% (154)
Supported living facility	7.3% (133)
Geriatric clinic	6.9% (125)
Private practice	5.6% (101)
Research centre	2.4% (44)
Other	14.4% (261)

Note. Participants could select >1 category; therefore, the total exceeds 100%.

**Table 2:** Awareness of relationship between risk factors and disordered sleep (%).

Related factor	Psychiatrist	Physician	Social worker	OT	PT	Nursing	Pharmacist	RT	Stat Sig.
Appetite	11.9*					33.0^∧^			*P* < 0.001
Falls		51.2^∧^					26.9*		0.006
Social withdrawal			37.8*					57.6^∧^	
Problem solving				39.8*				54.2^∧^	
Aggression					29.3*			61.0^∧^	*P* < 0.001
Depression	81.0^∧^		23.3*						0.003
Daytime sleepiness		88.9^∧^	70.3*						
Night wakefulness	82.1^∧^						62.7*		
Napping	76.2^∧^				58.8*				0.008
Medication		69.1^∧^				42.7*			*P* < 0.001
Cognitive decline	79.0^∧^							40.6*	*P* < 0.001
Comorbidity	67.9^∧^					33.4		25.4*	*P* < 0.001
Decreased mobility			51.4*		69.8^∧^				
Alcohol use		38.3^∧^						5.1*	*P* < 0.001
Smoking	14.3^∧^						1.5*		
Caregiver beliefs		21.0^∧^	3.4*						0.011

Key. ^∧^highest endorsement, *lowest endorsement, OT: occupational therapist, PT: physical therapist, RT: recreation therapist. Note. Insufficient sample of psychologists, respiratory technicians, and assistants for analysis.

**Table 3:** Awareness of relationship between comorbidity and disordered sleep (%).

Condition	Psychiatrist	Physician	Social worker	OT	PT	Nursing	Pharmacist	RT	Stat sig.
Allergies			8.1^∧^					1.7*	
Cardiovascular	61.9^∧^							30.5*	*P* < 0.001
Substance abuse	55.4^∧^							11.9*	*P* < 0.001
Endocrine disorder		16.0^∧^						1.7*	*P* < 0.001
Obesity	50.0^∧^			19.6*					*P* < 0.001
GI disorders	19.0*					32.1^∧^			
Infection							9.0*	35.6^∧^	*P* < 0.001
Pain	85.7^∧^		64.9*						*P* < 0.011
Neurological		60.5^∧^						35.6*	
Skin condition		25.9^∧^			4.0*				*P* < 0.001
Mental health	92.9^∧^				63.8*				*P* < 0.001
Pulmonary	50.0^∧^						28.4*		*P* = 0.006
Renal disorder	2.4*		16.2^∧^						*P* = 0.003
Rheumatic disease		24.7^∧^						10.2*	
Sensory deficit					7.5*			23.7^∧^	*P* < 0.001
Urological cond.		74.1^∧^						47.5*	*P* < 0.001

Key. ^∧^highest endorsement, *lowest endorsement, OT: occupational therapist, PT: physical therapist, RT: recreation therapist. Note. insufficient sample of psychologists, respiratory technicians, and assistants for analysis.
